# Utility and Accuracy of Primary and Secondary Ultrasonographic Signs for Diagnosing Acute Appendicitis in Pediatric Patients

**DOI:** 10.7759/cureus.3779

**Published:** 2018-12-27

**Authors:** Waseem A Mirza, Mujtaba Z Naveed, Kumail Khandwala

**Affiliations:** 1 Radiology, Aga Khan University, Karachi, PAK

**Keywords:** ultrasound, sonographic, pediatric, acute appendicitis, secondary signs

## Abstract

Introduction

Making an accurate diagnosis of acute appendicitis (AA) is vital to prevent the morbid complications associated with untreated AA. This is challenging in up to 30% of pediatric patients which is a significantly high number. Ultrasound (US) has been generally used as the initial mode of imaging in pediatric patients due to the lack of ionizing radiation. Given its variable accuracy, adjuvants such as secondary signs can be used to aid the radiologist in making an accurate diagnosis.

Materials and methods

Patients between the ages of two and sixteen years with acute abdominal pain suspicious for AA, who underwent right lower quadrant US between 2003 and 2016, were retrospectively identified. Corresponding computed tomography (CT) and histopathology findings were noted. Based on the presence of primary and secondary signs, results were classified into three groups to determine accuracy. Group 1 included all patients with a normal appendix or if the appendix was not visualized, no secondary signs were present. Group 2 patients were those in which the appendix was not clearly seen and they had one or more secondary signs of AA. Group 3 included all patients with primary signs of AA. The number of secondary signs and cases with perforated appendices were also correlated with sonographic accuracy.

Results

One thousand one hundred and fifteen patients met the inclusion criteria of which 29% had confirmatory AA. The positive appendectomy rate was 89% (337/380). Using a 3-category classification of US results, the sensitivity was 79%, specificity 97%, positive predictive value was 93%, negative predictive value was 91% and the overall accuracy was 91%. The presence of two or more secondary signs had a high likelihood of appendicitis. The perforation rate was 10% with the highest percentage seen in Group 2 patients.

Conclusion

Despite inescapable limiting factors, US should be used as first-line imaging for suspected appendicitis in pediatric patients especially since its accuracy rivals CT when the appendix is visualized. The use of secondary sonographic signs has solid potential to aid the radiologist in making an accurate diagnosis with our study demonstrating a proportional relationship between the number of secondary signs and the likelihood of true appendicitis. However, further investigation is needed to determine the individual accuracy of secondary signs and whether a certain combination of secondary signs has a higher association with appendicitis.

## Introduction

Appendicitis is the most frequently encountered acute condition requiring surgical management in children and adults [1-3]. In about 30% of pediatric patients, making an accurate diagnosis can be challenging [2-3]. Unfortunately, there is a fine line between sending someone for potentially unnecessary invasive surgery versus risking the detrimental complications of untreated appendicitis. However, due to the untreated risk of perforation, abscess formation, bowel obstruction, peritonitis and sepsis [3], up to 20% negative appendectomy rate is considered acceptable in adults and children [4-5].

In order to prevent misdiagnosis of acute appendicitis (AA), ultrasound (US) and computed tomography (CT) are being heavily relied upon to aid in making the correct diagnosis [3,6]. CT scan has better overall sensitivity and specificity for diagnosing AA compared to US [6-7], with documented sensitivity and specificity ranging from 95% to 97% and 94% to 97%, respectively [8-9]. The dilemma of using CT in pediatric patients stems from the exposure of ionizing radiation. Children are approximately 10 times more sensitive to ionizing radiation compared to adults [1,10], thus increasing their risk of developing cancer in the future [11-12]. Although magnetic resonance imaging (MRI) has shown similar results to CT with the advantage of not exposing the patient to ionizing radiation [1,13], its biggest drawback remains a relatively high cost, availability and prolonged acquisition times with the use of general anesthesia in the pediatric age group. Thus, US is the preferred first-line diagnostic imaging for evaluation of AA in pediatric patients [3-4,6].

US provides a non-invasive, readily available, and cost-effective way of diagnosing AA without exposing the patient to ionizing radiation. However, the accuracy of US has varied widely in the literature. This is secondary to the appendix visualization rate, which ranges from 40% to 89% [3,8,14-15]. US can be as accurate if not more as CT if the appendix is visualized. Factors that play a role in appendix visualization include operator experience (seasoned technologist, pediatric radiologist versus general radiologist), patient body habitus and anatomical position of the appendix.

Secondary signs (SS) have been claimed to be useful when the appendix is not visualized and AA is still suspected. These are anatomical descriptions of inflammation within the surrounding tissue caused by AA. There is a paucity of data as to how valuable SS can be in terms of type and number. Thus, the purpose of our study is to shed further light on the utility of US and its SS in diagnosing AA.

## Materials and methods

The study was approved by the Ethics Review Committee of our institute and the need for informed consent was waived. We conducted a retrospective review to determine the accuracy of US in the diagnosis of AA in the pediatric population and to evaluate the utility of SS in cases where the appendix was not visualized. The study was conducted at the Aga Khan University Hospital, a tertiary care center located in the heart of the largest metropolitan city in the country. Subjects aged from two to sixteen years who had undergone US right lower quadrant (RLQ) for acute abdominal pain with clinical suspicion for AA, were retrieved from the departmental database from June 15, 2003 to December 9, 2016.

Patients were excluded if they had primary and/or SS of appendicitis on US without CT or biopsy confirmation. Also excluded were patients whose indication was to evaluate an already known condition and those who were status post appendectomy. Patients with negative US RLQ (no primary and SS of AA) were included regardless of a confirmatory CT or biopsy. 

The graded compression technique described by Puylaert [16] (according to our departmental protocol) was used and all examinations were performed using Toshiba Xario (Toshiba Medical Systems Corporation, Japan) with 3.5-10 MHz probes. Board certified radiologists performed US during the day and third and fourth year (senior) radiology residents performed US overnight. All US images and reports were finalized by board certified radiologists with experience ranging from three to 26 years.

Data were transcribed from the hospital's medical record system. This included US findings with corresponding CT scan and histopathology results when present and patient demographics. US reports were retrospectively classified into three groups by the principal investigator: Group 1 included all patients with a normal appendix or if the appendix was not visualized, no SS were present. Patients were classified into Group 2 if the appendix was not clearly seen and they had one or more SS of AA. Group 3 included all patients with primary signs of AA. 

Primary signs of AA were defined as a blind-ending loop measuring greater than or equal to 6 mm in diameter, demonstrating any of the following features: non-compressibility, aperistalsis, increased wall thickness and vascularity. The following SS were recorded: free fluid (reports with mild free fluid were excluded as this is a normal finding), lymph node greater than or equal to 8 mm in diameter, echogenic fat in the RLQ, decreased peristalsis, omental thickening, RLQ collection, cecal thickening and appendicolith. CT and histopathology (biopsy) criteria were either positive (AA) or negative (normal appendix).

The diagnostic accuracy of US and SS for AA was determined using the standard epidemiological method of calculating the sensitivity, specificity, positive predictive value (PPV) and negative predictive value (NPV). The number of SS was correlated with the presence of AA. Statistical analysis was obtained using the Statistical Package for Social Sciences (SPSS) software version 21 (IBM, Armonk, NY, USA).

## Results

Our database yielded 1179 cases of suspected AA between the ages of two and sixteen years. Among these, 22 patients from Group 2 and 34 patients from Group 3 did not have CT or biopsy confirmation, and were therefore excluded. Another eight patients were excluded either because the appendix had been surgically removed or their indication for US was to evaluate another known entity. The final sample size was 1115 of which 714 were males and 401 were females. The mean age was 9.4 years. Three hundred and fifty eight (29%) patients had AA confirmed by CT and/or biopsy. Three hundred and eighty patients underwent appendectomy of which 89% (337/380) had AA and therefore, the negative appendectomy rate was 11% (43/380).

To calculate the accuracy of US RLQ for AA, we considered Group 1 to be negative and Group 2 and Group 3 as positive. Using this classification method, the sensitivity was 79%, specificity 97%, PPV was 93%, NPV was 91% and the overall accuracy was 91% (Table 1).

**Table 1:** Accuracy of ultrasound classification for acute appendicitis PPV: positive predictive value; NPV: negative predictive value

Parameter Percentage (Total number)	
Sensitivity	79% (283/358)
Specificity	97% (735/757)
PPV	93% (283/305)
NPV	91% (735/810)
Accuracy	91% (1018/1115)

Group 1 included patients in whom the appendix was normal or if the appendix was not seen, there were no primary or SS of AA (n=810, 73%). Among these, 735 (91%) did not have AA. There were 52 (5%) patients with SS in the absence of a visualized appendix in Group 2. Appendicitis was present in 38/51 (75%) patients and three patients had a negative appendectomy. In Group 3, there were 254 (19%) patients who had primary signs of AA. Out of the 254, 245 (96%) patients were confirmed to have AA. Among the 43 patients with a negative appendectomy, Group 3 had 11 (Table 2).

**Table 2:** Confirmed appendicitis (computed tomography + biopsy) and negative appendectomy (biopsy)

	Total	Group 1 (n=810) Normal appendix or appendix not seen, absent secondary signs	Group 2 (n=52) Appendix not seen, secondary signs present	Group 3 (n=253) Appendicitis
Appendicitis	358	75 (9%)	38 (75%)	245 (96%)
Negative appendectomy	43	29	3	11

A total of 68 SS were noted in the 52 patients of Group 2. The most frequently encountered SS was enlarged lymph node (n=21), and AA was present in 86% (18/21) of these patients. Echogenic fat in the right iliac fossa was the second most common SS and was seen in 15 patients, and 10 (67%) had AA. 10 patients had decreased peristalsis of which seven (70%) had AA. There were eight patients with free fluid exceeding physiological amounts, per the discretion of the radiologists, six (75%) of these patients had AA. A collection was visualized in seven patients of which five (71%) had AA. Cecal thickening/edema was present in three patients, all of whom had AA (100%). There was appendicolith in two patients, both had AA (100%), whilst omental thickening was also seen in two patients, only one had AA (50%) (Table 3).

**Table 3:** Accuracy of secondary ultrasound findings for appendicitis PPV: positive predictive value; NPV: negative predictive value; RLQ: right lower quadrant

Secondary Sign	Total	Appendicitis	Sensitivity	Specificity	PPV	NPV	Accuracy
Lymph nodes	21	18	19%	100%	86%	91%	91%
Echogenic fat (RLQ)	15	10	12%	99%	67%	91%	90%
Decrease peristalsis	10	7	9%	100%	70%	91%	90%
Free fluid (moderate to large)	8	6	7%	100%	75%	91%	91%
Collection	7	5	6%	100%	71%	91%	91%
Cecal thickening/edema	3	3	4%	100%	100%	91%	91%
Appendicolith	2	2	3%	100%	100%	91%	91%
Omental thickening	2	1	1%	100%	50%	91%	91%

There were 40/52 patients with only one SS, and AA was found in 28 (70%) of these patients. Two SS were seen in nine out of 52 patients of whom seven (78%) had AA. Three and four SS were seen only in two and one patients respectively, all of whom had AA (100%) (Table 4).

**Table 4:** Relationship between number of secondary signs and appendicitis

	Total	Appendicitis	Prevalence
One secondary sign	40	28	70%
Two secondary signs	9	7	78%
Three secondary signs	2	2	100%
Four secondary signs	1	1	100%

The overall perforation rate was 10% (n=36) of which 28% (10/36) were detected on US. There were five perforations in Group 1, Group 2 had eight, and Group 3 had the most with 23 (Table 5). There was one US RLQ that was false positive for perforation although the patient did have AA on biopsy.

**Table 5:** Utility of ultrasound for perforations US: ultrasound

	Total	Group 1 (n=810) Normal appendix or appendix not seen, absent secondary signs	Group 2 (n=52) Appendix not seen, secondary signs present	Group 3 (n=253) Appendicitis
Perforations	36	5	8	23
Perforation detected by US	10	0	3	7

## Discussion

Our study corroborates that US should be used as first-line imaging for suspected appendicitis in children and that SS has a strong potential to aid in making in the correct diagnosis [3,7]. The prevalence of AA in our study was 29% which is similar to other reported studies (25%, 32%, 34%). Our US accuracy of 91% for diagnosing AA in pediatric patients was also comparable with prior studies [3-4,7,14,17].

Despite our acceptable results, 91 patients were incorrectly diagnosed. Lack of appendix visualization is the most important factor. There are many factors that can contribute to lack of appendix visualization such as; imaging a spontaneously resolving appendicitis, inability to detect early AA, operator experience, patients’ physique; pain status and sensitivity, location of the appendix and high patient volume setups resulting in shorter scan times for locating the appendix [7,17]. Unfortunately, many of these factors are out of one's control which is also why surgeons sometimes overlook imaging results if they don't correlate with clinical parameters [18]. Based on our experience, if the appendix is not visualized and no SS are present, we believe that the patient should be managed operatively if there is high clinical suspicion [3].

The presence of appendicitis without primary or secondary signs was 9%, slightly higher compared to 7.1% obtained in the study by Estey et al. [3], whilst Wiersma et al. [7] had none but their sample size was 212 compared to our 1115. Despite this, our NPV was greater than 90% which is concordant with prior studies [14].

**Figure 1:**
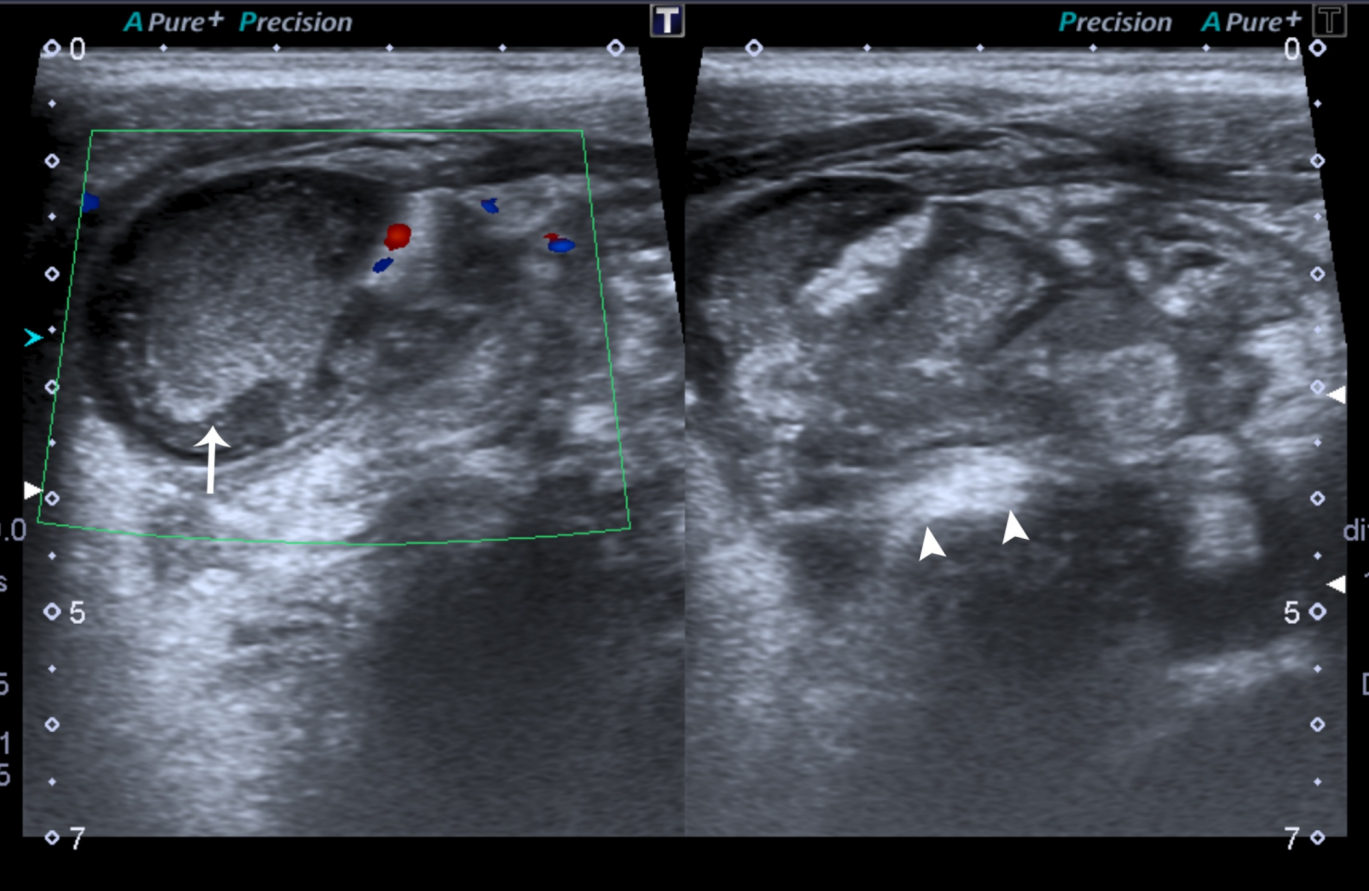
Sonographic images of a 10-year-old male A heterogenous collection with moving internal echoes (arrow) was noted in the right iliac fossa with echogenic inflammation of the surrounding fat (arrowheads). The appendix was not separately visualised. This patient had perforated appendicitis, which was confirmed on surgery.

In our study, only 4.7% (52) patients had SS in the absence of a visualized appendix. The prevalence of this group in the literature ranges from 3%-45% [3-4,14]. The major issue with SS is that they are not sensitive. Furthermore, most patients will only have one to two SS if any at all, and in our opinion, these patients pose the greatest diagnostic dilemma. However, in our study certain SS such as cecal thickening and the presence of appendicolith had a 100% PPV. RLQ echogenic fat, decreased peristalsis, free fluid and collection had a reasonable PPV ranging 67-75% (Figure 1). Although the presence of three or more SS is very uncommon [3,14] we felt appendicitis could be confidently diagnosed in this patient group (three patients - all had AA). 

The perforation rate among patients with AA was 10.1%. The highest percentage of perforations was found in Group 2 (20.1%), similar to Wiersma et al. [7]. This is because a perforated appendix is relatively difficult to visualize. Group 1 and Group 3 had a perforation rate of 6.7% and 9.4%, respectively.

Methods to standardize the US reporting system with the inclusion of SS have shown to improve patient management. A study conducted by Partain et al. demonstrated that the use of a standardized US reporting system coupled with increased reporting of SS resulted in a decreased use of CT and lowered the admission rate for observation of patients with suspected AA and equivocal US results [8]. Larson et al. proposed a five-category interpretive scheme based on appendix visualization, which allowed for more specific guidance for clinical and surgical management potentially reducing the number of negative appendectomies and providing superior confidence in the interpretation of cases where the appendix was not seen [19].

There are several limitations in our study. Firstly, it is a retrospective study and therefore carries associated limitations. Secondly, which we feel is the most problematic limitation and also seen in other similar studies, is the relatively low number of patients with SS. Additionally, there is no way of truly knowing whether a patient that presented with US was truly negative without histopathology. There is also a lot of variability in results due to factors such as operator experience. Hence, larger multicentric prospective studies are required ideally using standardized reporting algorithms to further determine how individual SS and a combination of SS correlate with AA.

## Conclusions

US should always be used as first line for the diagnosis of AA in pediatric patients. SS have the key potential to aid in making an accurate diagnosis when the appendix is not visualized. In our study, equivocal cases with the presence of three or more secondary signs had a high probability index for AA. However, more research in the form of a prospective study is needed for evaluation of how secondary signs either individually or collectively can further aid the radiologist to make an accurate diagnosis. 
